# Case report: schwannoma arising from the unilateral adrenal area with bilateral hyperaldosteronism

**DOI:** 10.1186/s12902-017-0225-z

**Published:** 2017-12-06

**Authors:** Naru Babaya, Yukako Makutani, Shinsuke Noso, Yoshihisa Hiromine, Hiroyuki Ito, Yasunori Taketomo, Kazuki Ueda, Hokuto Ushijima, Yoshifumi Komoike, Yuto Yamazaki, Hironobu Sasano, Yumiko Kawabata, Hiroshi Ikegami

**Affiliations:** 1 Department of Endocrinology, Metabolism and Diabetes, Kindai University Faculty of Medicine, 377-2 Ohno-higashi, Osaka-sayama, Osaka, 589-8511 Japan; 2 Department of Surgery, Kindai University Faculty of Medicine, 377-2 Ohno-higashi, Osaka-sayama, Osaka, 589-8511 Japan; 3 Department of Pathology, Tohoku University Graduate School of Medicine, 2-1 Seiryo-Machi, Aoba-ku, Sendai, Miyagi 980-8575 Japan

**Keywords:** Hyperaldosteronism, Incidentaloma, Paradoxical hyperplasia, Micronodular hyperplasia, Aldosterone-producing cell clusters

## Abstract

**Background:**

We report a rare case of a juxta-adrenal schwannoma that could not be discriminated from an adrenal tumor before surgical resection and was complicated by bilateral hyperaldosteronism. To the best of our knowledge, this is first case in which both a juxta-adrenal schwannoma and hyperaldosteronism co-existed.

**Case presentation:**

A 69-year-old male treated for hypertension was found to have a left supra-renal mass (5.8 × 5.2 cm) by abdominal computed tomography. His laboratory data showed that his plasma aldosterone concentration (PAC) was within the normal range, but his plasma renin activity (PRA) was reduced, resulting in an increased aldosterone/renin ratio (ARR). Load tests of captopril or furosemide in the standing position demonstrated autonomous aldosterone secretion and renin suppression. Adrenal venous sampling (AVS) with ACTH stimulation indicated bilateral hypersecretion of aldosterone. A left supra-renal tumor was resected because of the possibility of malignancy and was found to be a benign schwannoma arising from the juxta-adrenal region together with an adrenal gland. The dissected left adrenal gland was morphologically hyperplastic in the zona glomerulosa, but was immunohistochemically negative for CYP11B2 (aldosterone synthase). Multiple CYP11B2-positive adrenocortical micronodules were detected in the adrenal gland, indicating micronodular hyperplasia. Although bilateral aldosteronism was indicated by AVS before the operation, the PRA, PAC and ARR values were within their respective reference ranges after resection of the unilateral tumor, suggesting that the slight increase in hormone secretion from the remaining right-sided lesion could not be detected after resection.

**Conclusion:**

A clinical and morphologic diagnosis of juxta-adrenal schwannoma is difficult, particularly in a case of hyperaldosteronism, as shown in this case. These data suggest the complexity and difficulty diagnosing adrenal incidentaloma.

## Background

A schwannoma is a benign, encapsulated and slowly progressive tumor originating from the nerve sheath. It occurs predominantly in the head and neck region as well as in the flexor surfaces of the upper and lower extremities [1]. Retroperitoneal schwannomas are rare [2, 3]. In particular, a visceral schwannoma in the adrenal region is extremely rare. Juxta-adrenal and adrenal schwannomas may cause non-specific clinical symptoms as symptoms and laboratory data specific to this particular disease have not been defined. The low incidence and asymptomatic nature of the disease with no hormonal production make a definitive diagnosis of schwannoma in the adrenal region difficult, leading to misinterpretation of the mass as an adrenal adenoma or cancer [2]. Surgery is typically required to establish a diagnosis and for treatment.

We treated a 69-year-old male patient with a juxta-adrenal schwannoma that could not be diagnosed preoperatively.

## Case presentation

**Fig. 1:**
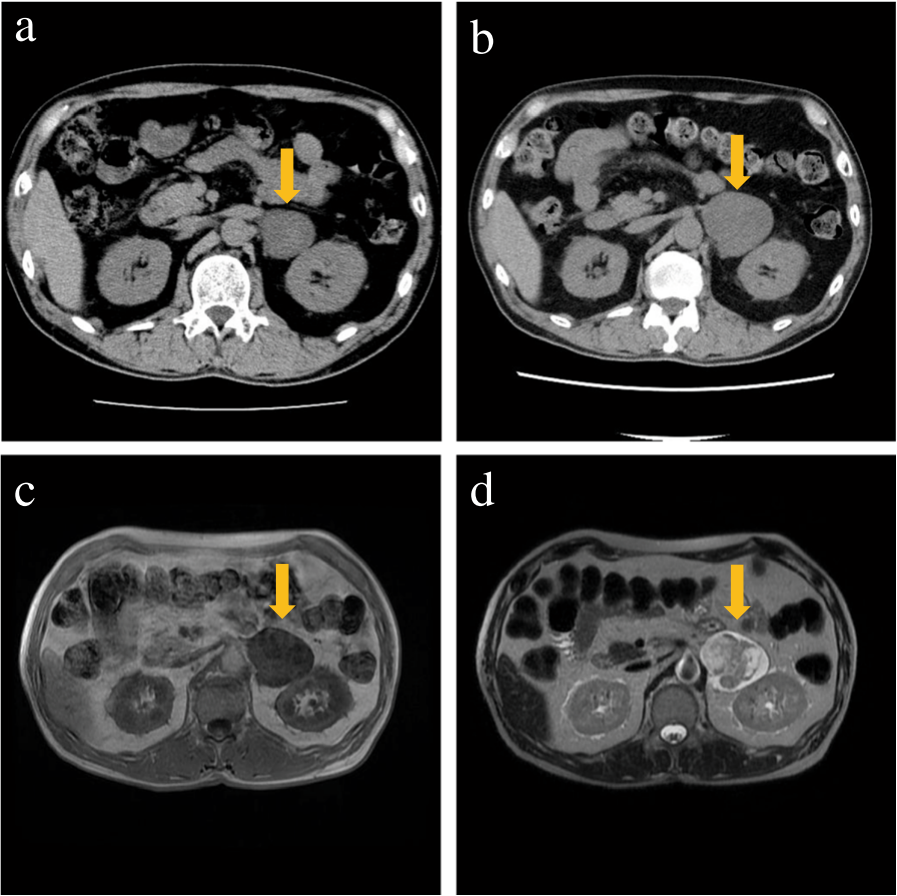
CT of the left adrenal area showing a mass (4.5 × 3.8 cm) when the patient was 60 years old (**a**) and a larger mass (5.8 × 5.2 cm) when he was 69 years old (**b**). MRI of the left adrenal area on T1-WI (**c**) and T2-WI (**d**) when the patient was 69 years old. Yellow arrows show the lesions of the adrenal area

Our patient is a 69-year-old Japanese man. At the age of 55 years, he underwent a medical assessment for occasional left abdominal pain. A left suprarenal mass was discovered by abdominal computed tomography (CT), and he was referred to our hospital. The tumor was 4 cm in size at that time, and no hormonal production had been detected (plasma renin activity (PRA), 0.5 ng/mL/h; plasma aldosterone concentration (PAC), 105 pg/mL; aldosterone/renin ratio (ARR), 210; the load test of furosemide in the standing position (PRA of 2.3 ng/mL/h, PAC of 236 pg/mL, ARR of 102.6); normal catecholamine and cortisol levels). Consequently, a diagnosis of a non-functional adrenal tumor was determined. The patient did not want to undergo surgery and was followed without surgical excision. The follow-up at our hospital continued for 5 years, and the tumor size (4.5 × 3.8 cm) did not appear to be increasing (the CT scan from the final visit is shown in Fig. 1a).

At the age of 65 years, he experienced an acute myocardial infarction and was taken to another hospital. Afterwards, he periodically visited the hospital for cardiac function assessments and hypertension treatment. At the age of 69 years, he was referred to our hospital because of a left suprarenal mass identified on abdominal CT (Fig. 1b) as part of a regular medical examination for ischemic heart disease and hypertension. He was admitted to our hospital for a detailed evaluation because the tumor had increased in size (5.8 × 5.2 cm). On admission, he was apparently in good health with no symptoms. He had been taking an antihypertensive drug (Amlodipine besylate 2.5 mg/day). A physical examination revealed no significant findings. His blood pressure was 136/76 mmHg. The laboratory data are shown in Table 1. The complete blood count and blood biochemistry tests were within normal ranges. The endocrinological data were within the normal range except for abnormal PRA and ARR values. The PAC was within the normal range (66 pg/mL), but PRA was reduced (0.2 ng/mL/h), resulting in an increased ARR (330).

**Table 1:** Laboratory data

Peripheral blood			Endocrinological data			Load test		
WBC	5630/mm^3^	(3300–8600)	[plasma]			[captopril]		
RBC	410 × 10^4^/mm^3^	(435–555)	Epinephrine	< 5 pg/mL	(0–100)	Renin	0 min	0.3 ng/mL/h
Hb	13.0 g/dL	(13.7–16.8)	Norepinephrine	69 pg/mL	(100–450)		60 min	< 0.1 ng/mL/h
Ht	38.9%	(40.7–50.1)	Dopamine	13 pg/mL	(0–20)		90 min	< 0.1 ng/mL/h
Plt	13.8 × 10^4^/mm^3^	(15.8–34.8)	Renin	0.2 ng/mL/h	(0.3–2.9)	Aldosterone	0 min	84.7 pg/mL
			Aldosterone	66 pg/mL	(29.9–159)		60 min	77.1 pg/mL
Biochemical data			ARR	330	(< 200)		90 min	72.7 pg/mL
T.P.	6.6 g/dL	(6.6–8.1)	ACTH	24.5 pg/mL	(7.2–63.3)			
Alb	4.2 g/dL	(4.1–5.1)	Cortisol	11.1 μg/dL	(6.2–19.4)	[furosemide with upright position]		
T.Bil	0.8 mg/dL	(0.4–1.5)	DHEA-S	100 μg/dL	(24–244)	Renin	0 min	0.3 ng/mL/h
AST	18 U/L	(13–30)					30 min	0.5 ng/mL/h
ALT	15 U/L	(10–42)	[urine]				60 min	0.7 ng/mL/h
LDH	207 U/L	(124–222)	Epinephrine	5.5 μg/day	(3.4–26.9)		120 min	0.9 ng/mL/h
ALP	149 U/L	(106–322)	Norepinephrine	97.9 μg/day	(48.6–168.4)	Aldosterone	0 min	70.1 pg/mL
rGTP	20 U/L	(13–64)	Dopamine	932.6 μg/day	(365–961.5)		30 min	154 pg/mL
BUN	16 mg/dL	(8–20)	Aldosterone	4.7 μg/day	(0–10)		60 min	177 pg/mL
Crea	0.84 mg/dL	(0.65–1.07)	Cortisol	45.3 μg/day	(11.2–80.3)		120 min	203 pg/mL
Na	140 mEq/L	(138–145)						
K	4.0 mEq/L	(3.6–4.8)						
Cl	108 mEq/L	(101–108)						
Glu	97 mg/dL	(73–109)						
T.chol	153 mg/dL	(142–220)						
HbA1c	5.8%	(4.9–6.2)						

Reference ranges are in parentheses. The judgment criteria of the load tests are as reported in [4]

To evaluate autonomous secretion of aldosterone and suppression of renin, load tests of captopril or furosemide in the standing position were carried out according to guidelines [4]. In Japan, if the ARR is high, at least 2 of 3 confirmation tests are recommended to make a definitive diagnosis (load tests of captopril or furosemide in the standing position, or saline) before attempting localization by adrenal vein sampling (AVS) [4]. No increase in renin activity was observed in the load tests, indicating autonomous secretion of aldosterone.

**Fig. 2:**
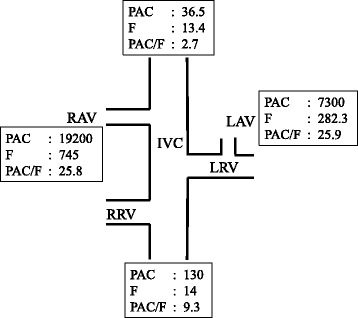
Adrenal venous sampling with ACTH stimulation. The PAC and serum cortisol levels in venous samples were selectively collected with a catheter. PAC: plasma aldosterone concentration (pg/mL). F: cortisol (μg/dL). IVC: inferior vena cava. LAV: left adrenal vein. LRV: left renal vein. RAV: right adrenal vein. RRV: right renal vein

AVS with ACTH stimulation indicated that the PAC in both adrenal veins was markedly high (Fig. 2). The PAC/cortisol ratio in both adrenal veins did not show laterality. These data demonstrate that both adrenal glands exhibited high and autonomous secretion of aldosterone, which is usually diagnosed as idiopathic hyperaldosteronism (IHA).

On CT scan (Fig. 1b), the left suprarenal mass was mostly circumscribed with a partial irregularity, and it was a homogenous mass (20~40 HU) with a density similar to that of the kidney (30~50 HU). The imaging diagnosis of the CT scan by the radiologist indicated an adrenal tumor. On magnetic resonance imaging (MRI), the tumor showed a low signal intensity on T1-weighted images (WI) (Fig. 1c) and heterogeneous signal intensity on T2-WI (Fig. 1d). Together with the results of MRI, the radiologist suggested that the tumor was a schwannoma.

**Fig. 3:**
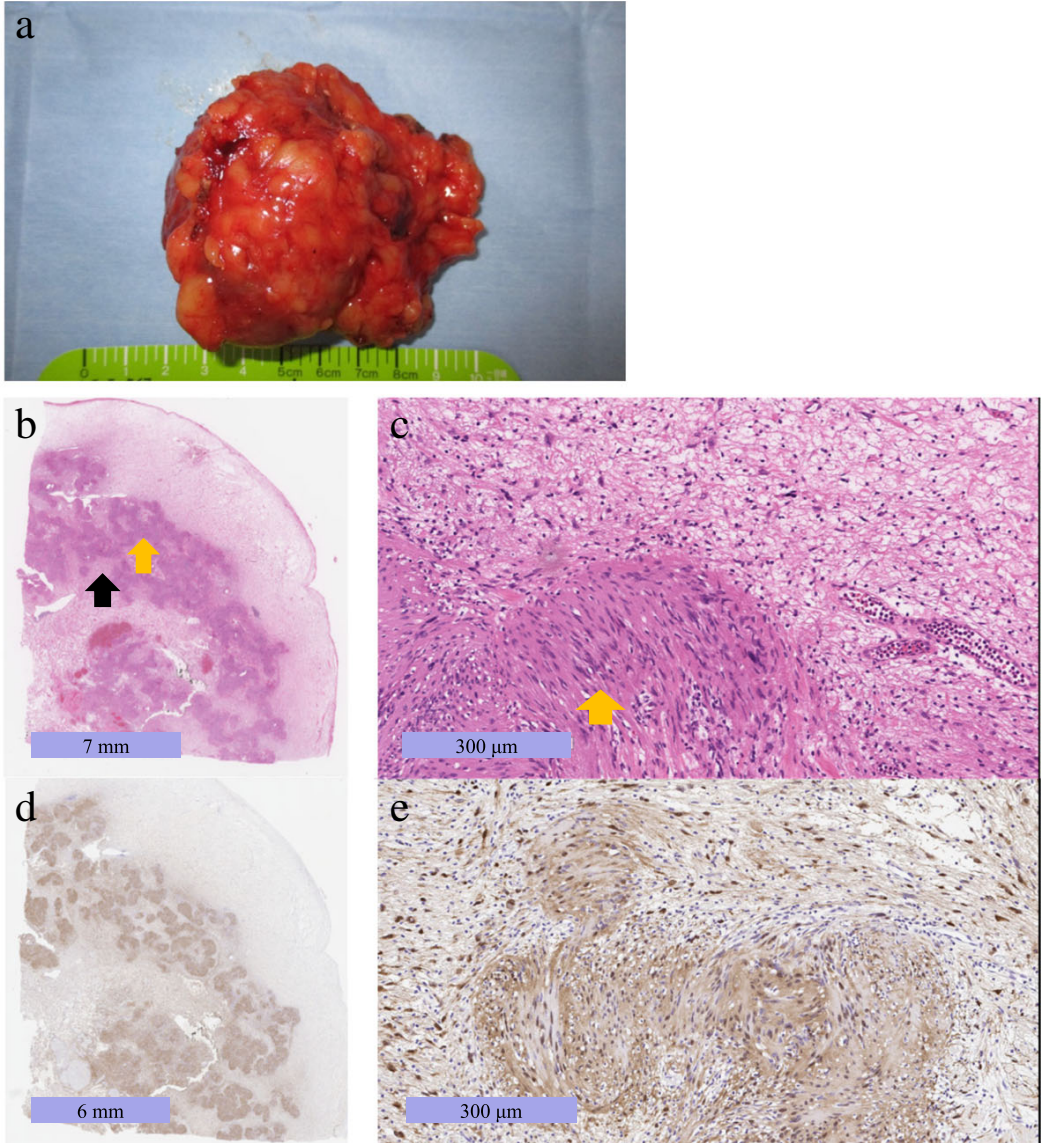
Macroscopic appearance (**a**) and photomicrograph (**b**-**e**) of the resected tumor. **b** The tumor was composed of a high cellular density area (Antoni A, yellow arrow) and low cellular density area (Antoni B, black arrow) (HE stain). **c** The Antoni A area included spindle-shaped cells with nuclear palisading (yellow arrow) (HE stain). **d**, **e** Immunohistochemical labeling in S-100. HE: Hematoxylin and eosin

Surgery was performed because of the possibility of malignancy, and a left supra-renal tumor was resected. On gross pathological examination (Fig. 3a), a clearly demarcated tumor (6.5 × 4.8 × 4.2 cm, 111 g) was observed with the adrenal gland and fat tissue. Histologically, the tumor was a typical schwannoma (Fig. 3b-e) and consisted of two different patterns of Antoni A and B components [5] without defined nuclear atypias. The Antoni A area had a high cellular density component characterized by closely packed spindle-shaped tumor cells with nuclear palisading, forming Verocay bodies, whereas the Antoni B area had a relatively low cellular density component with myxoid stroma. According to the immunohistochemical examination, the spindle-shaped tumor cells were strongly and diffusely positive for S-100 (Fig. 3d, e). Based on these histological findings, the tumor was diagnosed as a typical benign schwannoma.

**Fig. 4:**
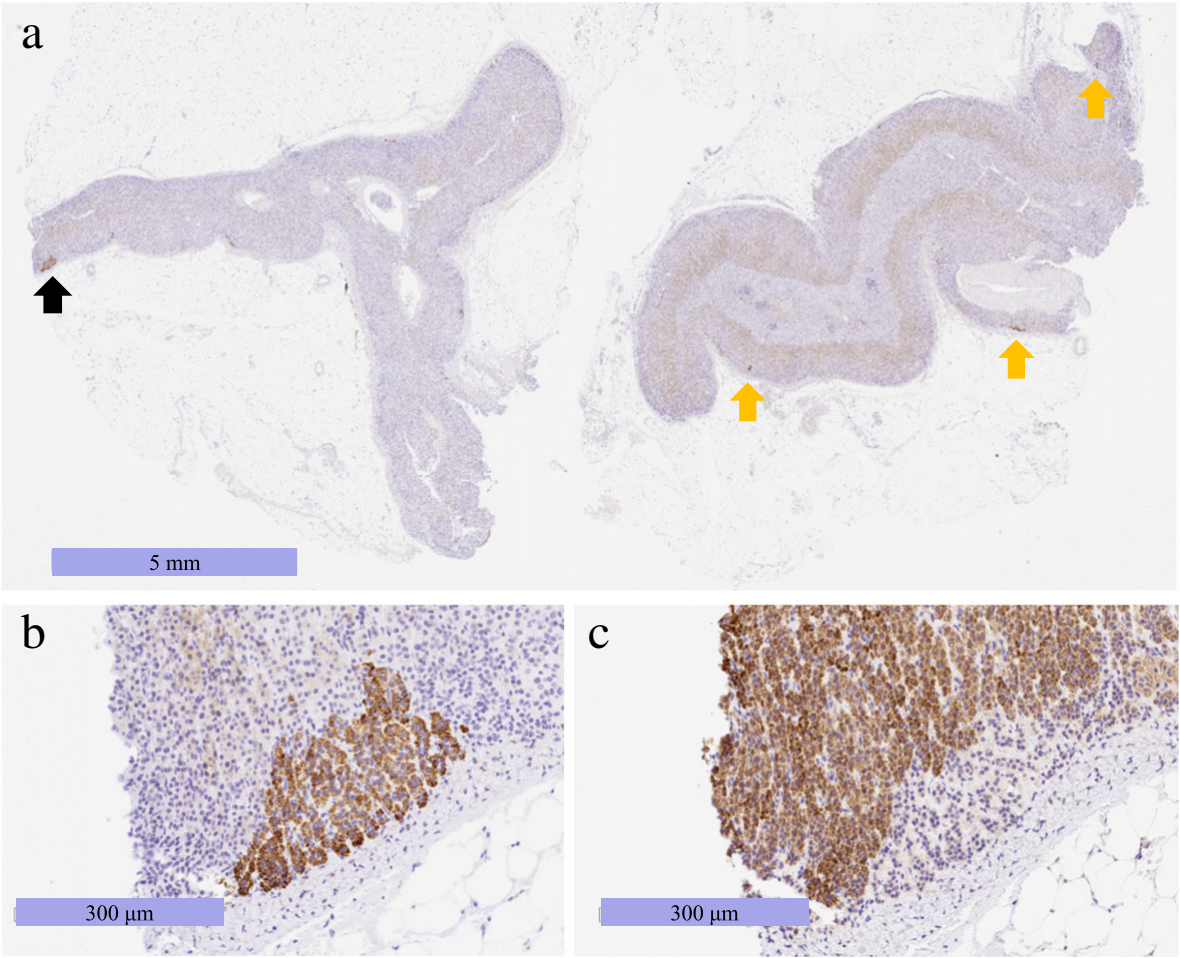
Photomicrograph of the resected left adrenal gland. **a** Immunohistochemical labeling in CYP11B2. Although the zona glomerulosa was almost negative for CYP11B2, several nodules were strongly positive (black and yellow arrows). **b**, **c** The CYP11B2-positive nodule indicated by a black arrow in Fig. 4a was magnified. Immunohistochemical labeling in CYP11B2 (**b**) and in CYP11B1 (**c**) CYP11B2: cytochrome P450 family 11 subfamily B member 2, CYP11B1: cytochrome P450 family 11 subfamily B member 1

Although the patient’s abdominal mass was a schwannoma adjacent to the adrenal gland, the preoperative examination could not detect whether the lesion was responsible for autonomous aldosterone hypersecretion. Therefore, we performed a histological analysis of the adrenal gland (Fig. 4). The dissected left adrenal gland was morphologically hyperplastic in the zona glomerulosa, but was immunohistochemically negative for CYP11B2 (aldosterone synthase [6, 7]), demonstrating “paradoxical hyperplasia” [7, 8] (Fig. 4a). Multiple CYP11B2-positive adrenocortical micronodules were detected in the resected adrenal gland (Fig. 4a, b), and these micronodules were negative for CYP11B1 (Fig. 4c). These histological findings demonstrated that the lesion responsible for autonomous aldosterone hypersecretion in the present case was derived from unilateral adrenocortical micronodules [7] rather than diffuse hyperplasia (DH) [7].

The postoperative course of the patient was uneventful. The patient is presently well and has shown no signs of recurrence of the schwannoma as of 6 months after the operation. His blood pressure is 110–130/70–90 mmHg with the administration of an antihypertensive drug (Amlodipine besylate 2.5 mg/day). His blood biochemistry data, including potassium, are within the normal range. His endocrinological data are also within the normal range, including the PAC (25.5 pg/mL), PRA (0.5 ng/mL/h) and ARR (51.0).

## Discussions

A schwannoma is usually a benign tumor emerging from a peripheral nerve sheath. A schwannoma in the adrenal gland is very rare [1, 9] and typically originates from the adrenal medulla. High levels of urinary catecholamine have been reported in several cases [3, 10]. Schwannomas of the retroperitoneum, especially the juxta-adrenal region, may be misdiagnosed as primary adrenal lesions [2] because of their shape. The remarkable aspect of our case is that aldosteronism was present in addition to the tumor, which led us the misdiagnosis of a primary adrenal tumor. Because of the tumor’s large size (5.8 × 5.2 cm) and slow growth over several years, surgical excision was chosen based on the recommendation that surgical removal should be considered for non-functional adrenal masses larger than 4 cm [5, 11]. Retroperitoneal nerve sheath tumors can be malignant and are potentially fatal, so it is important to remove retroperitoneal tumors completely [5].

In this case, the co-occurrence of a schwannoma and aldosterone-producing micronodules is considered to be incidental, but the possibility that humoral factors secreted from the schwannoma may have affected aldosterone secretion cannot be fully excluded. No case of a schwannoma and concurrent bilateral autonomous aldosterone-producing lesions in the same patient has been reported in the literature, including schwannomas arising from places other than the retroperitoneum. However, the etiologies of these non-neoplastic autonomous aldosterone-producing lesions in the adrenal gland remain unknown, and further investigations are required for clarification.

After the operation, the patient had normal blood pressure with the administration of an antihypertensive drug (Amlodipine besylate 2.5 mg/day). His PRA, PAC and ARR values were within the reference ranges even though bilateral aldosteronism was indicated by AVS before the operation, possibly because slight changes in hormone secretion from the remaining right-sided lesion cannot be detected in the peripheral blood. A careful follow-up of the hormone levels and a pathological examination of a right-sided adrenal lesion, if present, are needed to clarify this supposition. Yamazaki et al. reported that all bilateral cases of aldosteronism have similar pathological features in both the right and left adrenals (18 out of 18 cases) [7]. Therefore, we suppose that the right adrenal gland may have unilateral adrenocortical micronodules and will need to be followed carefully.

## Conclusions

Preoperative diagnosis of a juxta-adrenal schwannoma is difficult, especially in a case with hyperaldosteronism. To the best of our knowledge, this is first case in which both a juxta-adrenal schwannoma and hyperaldosteronism co-existed, demonstrating the complexity and difficulty of the diagnosis of adrenal incidentaloma.

## Abbreviations

ARR: Aldosterone/renin ratio
AVS: Adrenal venous sampling
CT: Computed tomography
DH: Diffuse hyperplasia
HE: Hematoxylin and eosin
IHA: Idiopathic hyperaldosteronism
MRI: Magnetic resonance imaging
PAC: Plasma aldosterone concentration
PRA: Plasma renin activity
WI: Weighted images
